# Krüppel-like factors in cancer progression: three fingers on the steering wheel

**DOI:** 10.18632/oncotarget.1456

**Published:** 2013-11-30

**Authors:** Ridha Limame, Ken Op de Beeck, Filip Lardon, Olivier De Wever, Patrick Pauwels

**Affiliations:** ^1^ Center for Oncological Research (CORE), University of Antwerp, Universiteitsplein 1, Antwerp, Belgium; ^2^ Department of Medical Genetics, University of Antwerp and Antwerp University Hospital, Universiteitsplein 1, Antwerp, Belgium; ^3^ Laboratory of Experimental Cancer Research, Department of Radiotherapy and Experimental Cancer Research, Ghent University Hospital, De Pintelaan 185, Ghent, Belgium; ^4^ Laboratory of Pathology, Antwerp University Hospital, Wilrijkstraat 10, Edegem (Antwerp), Belgium

**Keywords:** Krüppel-like factor, carcinoma, EMT, invasion, metastasis, pluripotency

## Abstract

Krüppel-like factors (*KLF*s) comprise a highly conserved family of zinc finger transcription factors, that are involved in a plethora of cellular processes, ranging from proliferation and apoptosis to differentiation, migration and pluripotency. During the last few years, evidence on their role and deregulation in different human cancers has been emerging. This review will discuss current knowledge on Krüppel-like transcription in the epithelial-mesenchymal transition (EMT), invasion and metastasis, with a focus on epithelial cancer biology and the extensive interface with pluripotency. Furthermore, as *KLF*s are able to mediate different outcomes, important influences of the cellular and microenvironmental context will be highlighted. Finally, we attempt to integrate diverse findings on *KLF* functions in EMT and stem cell biology to ft in the current model of cellular plasticity as a tool for successful metastatic dissemination.

## 1 The SP/KLF-family of transcription factors

Specificity proteins (*SP*) and Krüppel-like factors (*KLF*s) are collectively referred to as the *SP1-like/KLF* or *SP/KLF* family of transcription factors. *SP1* was first identified in the early 1980s as a protein that was able to bind GC- and related GT-rich regions or CACCC elements in the SV40 promoter and, therefore, could serve as a transcriptional regulator [1]. Several *SP*-like factors have been found since and, to date, this subfamily contains 9 members (*SP1* – *9*). The DNA-binding region of *SP*s consists of three highly conserved Cys_2_/His_2_ zinc fingers, localized near the C-terminal end of the protein [2]. The interfinger linking sequences, called “H/C links”, containing a stretch of seven amino acids also show a high degree of conservation (TGE(R/K)P(Y/F)X) within the family and between species [3]. The presence of this typical structure in the *Drosophila melanogaster* gap gene Krüppel [4] has given rise to the association with the *Krüppel-like* part of this transcription factor family [5].

**Fig 1:**
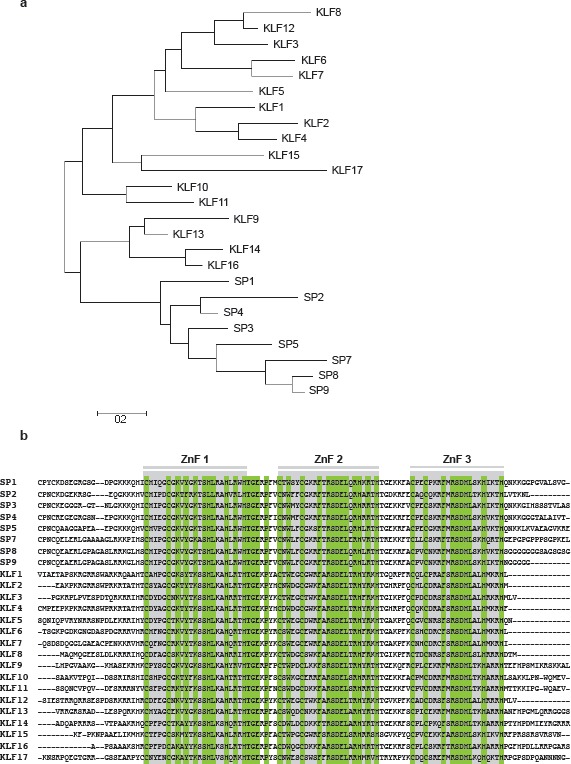
**a).** Phylogenetic tree resulting from a molecular phylogenetic analysis, based on the protein sequences of all human *SP*/*KLF* members using the Mega5 (v5.1) built-in Maximum Likelihood method and Nearest Neighbor-Joining algorithm [150]. b). Amino acid sequence alignment of the zinc finger C-terminal region of all known *SP*s and *KLF*s, as determined by ClustalW using Mega5 (v5.1) software. Indicated are the three separate zinc finger sequences (grey boxes, ZnF) and conserved amino acids (green)

The first mammalian homologue to *Drosophila* Krüppel was discovered in a murine erythroleukemic cell line [6] and named *E-KLF* (Erythroid Krüppel-like factor, *KLF1*). This factor was shown to trans-activate ß-globin expression by binding the CACCC element within its promoter [7]. Loss-of-function studies demonstrated that homozygous *E-KLF*^−/−^ mice developed fatal ß-thalassaemia during early fetal liver erythropoiesis [8] Numerous closely related human proteins have been identified since and collectively named Krüppel-like factors (*KLF*s), preceded by an index letter of the tissue or origin of enriched expression (*X-KLF*, Table 1 and Fig 1a).

**Table 1:** Classification and summary of the *SP/KLF* family in humans

HGNC	Alias	Gene accession #	UniProt_KB entry #	# amino acids	Molecular weight (kDa)
SP1	TFSP1	BC062539	P08047	785	80.7
SP2	KIAA0048	NM_003110	Q02086	613	64.9
SP3	SPR-2	AY070137	Q02447	781	81.9
SP4	SPR-1,HF1B, MGC130008, MGC130009	NM_003112	Q02446	784	82
SP5		AB096175	Q6BEB4	398	42
SP7	OSX	BC113613	Q8TDD2	431	45
SP8		BC038669	Q8IXZ3	490	48.7
SP9	ZNF990	NM_001145250	P0CG40	484	48.9
					
KLF1	E-KLF	JX877554	Q13351	362	38.2
KLF2	L-KLF	EF078888	Q9Y5W3	355	37.4
KLF3	B-KLF, TEF-2	NM_016531	P57682	345	38.8
KLF4	G-KLF, EZF	DQ658241	O43474	513	54
KLF5	I-KLF, C-KLF, BTEB2	AF287272	Q13887	457	50.8
KLF6	BCD1, COBEP, CBPB, ST12, GBF	AF284036	Q99612	283	31.9
KLF7	U-KLF		O75840	302	33.4
KLF8	BKLF3, ZNF741	NM_007250	O95600	359	39.3
KLF9	BTEB, BTEB1	NM_001206	Q13886	244	27.2
KLF10	TIEG, TIEG1, EGRα	NM_005655	Q13118	480	52.6
KLF11	F-KLF, TIEG2, MODY7		O14901	512	55.1
KLF12	AP2rep, HSPC122		Q9Y4X4	402	44.2
KLF13	BTEB3, NSLP1, RFLAT-1	NM_015995	Q9Y2Y9	288	31.2
KLF14	BTEB5, SP6, EPFN	DQ534757	Q8TD94	323	33.1
KLF15	K-KLF	NM_014079	Q9UIH9	416	44
KLF16	BTEB4, NSLP2, DRRF	NM_031918	Q9BXK1	252	25.4
KLF17	ZNF393	NM_173484	Q5JT82	389	42.6
					

Ablebbreviations: AP2rep AP2 repressor, B basic, BCD B-cell derived protein, BTEB basic transcription element binding, C colon, COBEP core promoter element binding protein, CPBP core promoter binding protein, DRRF dopamine receptor regulating factor, E erythroid, EGRα early growth response gene α, EPFN epiprofin, EZF epithelial zinc finger, F embryonic/fetal ß-like globin gene-activating, G gut, GBF GC-rich binding factor, I intestinal, K kidney, L lung, MODY7 maturity-onset diabetes of the young 7, NSLP Novel SP1-Like Protein, OSX osterix, RFLAT RANTES factor of late activated T-lymphocytes, SP specificity protein / SV40-promoter protein, ST suppressor of tumorigenicity, TFSP transcription factor SP, SPR SP1-related factor, TEF transcriptional enhancer factor, TIEG TGFß-inducible early gene, U ubiquitous, Z(N)F zinc finger

Sources: HUGO Gene Nomenclature Committee (www.genenames.org) and [9]. Protein molecular weights were retrieved from The Human Protein Atlas (www.proteinatlas.org) [149].

The homology between *SP/KLF* proteins is mainly restricted to the zinc finger and linker domains, situated at the C-terminal, and underlines the importance of this structure in transcription biology (Fig 1b). The major difference distinguishing *SP*s from *KLF*s is the absence of a Buttonhead box CXCPXC preceding the triple zinc finger region in the latter [9]. Furthermore, several *SP/KLF*s (*KLF1*, *2*, *4*, *9*, *13*, *16*) share a nuclear localization signal (NLS), necessary for post-translational transport towards the nucleus [10]. More variability exists within the N-terminal regions of *KLF*s, containing both activator and/or repressor domains that interact with specific coactivators and -repressors, providing unique functions to each family member.

As of currently, the *KLF* family of transcription factors comprises 17 identified members (*KLF1* - *17*) with diverse regulatory roles in differentiation, survival, proliferation and development. To avoid confusion because of alternative names, a straightforward *SP/KLF* nomenclature has been generated by the Human Genome Organization Gene Nomenclature Committee (HGNC, www.genenames.org) in which all *SP*s and *KLF*s have been numbered sequentially in order of their discovery (Table 1) [5,9]. This nomenclature will be followed throughout this review.

For reasons of conciseness, this review will further handle current knowledge on the *KLF* subfamily within the epithelial-mesenchymal transition (EMT), carcinoma progression and metastasis, added with uncovered roles in induced pluripotency and self renewal biology. More extended structural and functional information regarding *SP*s can be found in previous reviews [11,12].

## 2 KLFs in EMT and invasion

### 2.1 EMT

EMT constitutes a transdifferentiation program whereby cells shift from an apical-basal to front-back polarity. The existence of EMT was first established within embryonic development [13]. This transition characteristically involves the loss of adherens junctions, typically E-cadherin (*CDH1*), that guarantee the lateral cell-cell contacts in epithelial layers. In parallel, desmosomes, cytoplasmic ß-catenin (*CTNNB1*), tight junctions (claudins, occludins and ZO-1/*TJP1*) and epithelial cytokeratins (CK18/*KRT18*) become downregulated. On the other hand, the expression of vimentin (*VIM*), part of the mesenchymal intermediate cytoskeleton, is induced together with N-cadherin (*CDH2*). In addition, the increased deposition of cellular fibronectin (*FN1*) and the subsequent activation of integrins facilitate cell migration and extracellular matrix (ECM) invasion [14,15].

EMT provides a mechanism that enables cancer cells to invade individually into the surrounding stroma. In addition, the morphotype switch has been demonstrated to be reversible, with the existence of mesenchymal-epithelial transition (MET) as an inverse mechanism, necessary during metastatic colonization [13,16].

**Fig 2:**
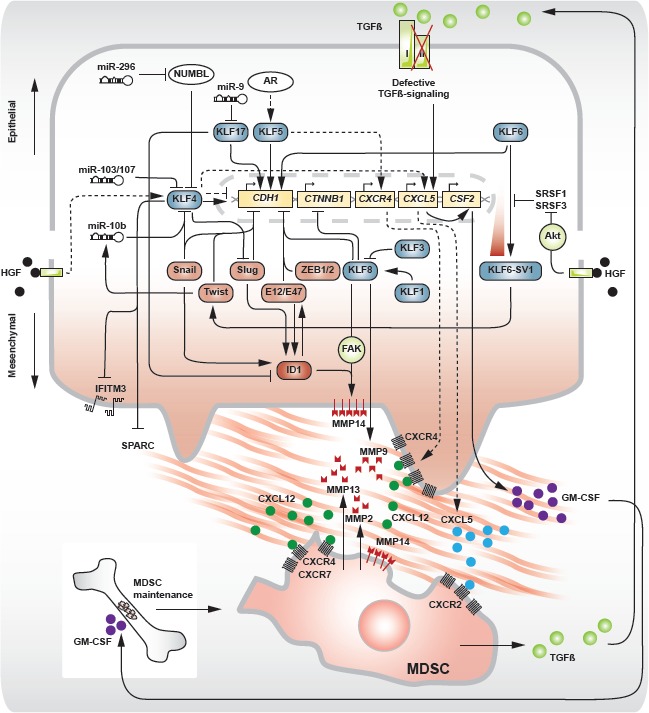
Involvement of different *KLFs* in the molecular circuitry of EMT and invasion in a single cell and interaction with a recruited myeloid-derived suppressor cell (MDSC) Upper and lower half of the main image represent epithelial and mesenchymal states respectively. Arrows and perpendicular symbols indicate promoting and inhibitory interactions respectively. Dotted arrows indicate pro-invasive interactions of *KLF4* and *KLF5*. Boxes: *KLF* (blue), EMT master transcription factors (red), pro-metastatic factors (deep red), genes (yellow), kinase (green). Filled circles represent different secreted chemokines/growth factors, as indicated.

Several master transcription factors have been identified in conferring EMT (Fig 2): Snail (*SNAI1*) [17,18], Slug (*SNAI2*) [19], Twist (*TWIST1*) [20], ZEB1/δEF1 (*ZEB1*) [21,22], ZEB2/SIP1 (*ZEB2*) [23], E12/E47 (*TCF3*) [24], Hey1 (*HEY1*) [25] and HMGA2 (*HMGA2*) [26]. Most of these factors primarily share the direct repression of *CDH1* through binding of E-boxes in its promoter.

*KLF8* is a potent inducer of EMT through *CDH1* repression [27]. *KLF8* was shown to directly bind to a GT box within the *CDH1* promoter, hereby uncovering the presence of another consensus element that is targeted by the distinct family of *KLF* zinc finger transcription factors, next to the known triple E-box motif. As the DNA binding zinc finger regions are highly conserved between *KLF*s, similar target sequences may be recognized by different members. The variable nature of the N-terminal domains, on the other hand, may give rise to opposite *trans*-regulatory effects. Despite the ambiguous reporting on the tumor suppressing yet oncogenic roles of *KLF4* in epithelial cancer biology (see *invasion and metastasis* and Fig 1), its function in EMT/MET has increasingly become clear. In cancer-related EMT as well as in EMT processes that contribute to the reprogramming route, *KLF4* is a potent inducer of epithelial differentiation and antagonizes the switch to a mesenchymal phenotype. Transcriptional regulation by *KLF4* increases the expression of *CDH1* and forces adult human fibroblasts into an epithelial state that proved a prerequisite for successful reprogramming to pluripotency [28]. Indeed, in MCF-10A normal mammary epithelium, *KLF4* was shown to activate *CDH1* transcription through binding of CACCC consensus sequences in the proximal promoter of the *CDH1* gene [29]. *KLF4* silencing resulted in a cadherin switch (loss of *CDH1* with concomitant gain of *CDH2* [30]) and a decrease of cytosolic ß-catenin. Consequently, overexpression of *KLF4* in the metastatic MDA-MB-231 breast cancer cell line dramatically increased *CDH1* and *KRT18* expression, indicating the restoration of an epithelial phenotype and loss of metastasis. The mechanism of EMT impairment by *KLF4* can be explained by its repressive action on the EMT transcription factors *SNAI1* [31] and *SNAI2* [28,32]. Conversely, in EMT-induced colon cancer cells, Snail was shown to repress the expression of *KLF4* [33]. This finding fits within a previously described role of *KLF4* in specific aspects of epithelial cell differentiation [34] and provides a rationale for loss of this factor in cancer. Direct binding sites for *KLF4* have been detected in the promoter sequences of vimentin (*VIM*), VEGF-A (*VEGFA*), endothelin-1 (*EDN1*) and JNK-1 (*MAPK8*), next to E-cadherin (*CDH1*), N-cadherin (*CDH2*) and *CTNNB1*, indicating a central role for *KLF4* within the EMT program [35]. These findings can at least partly explain the downregulation of *KLF4* as reported for several epithelial cancer types and the inverse correlation of *KLF4* expression with clinical outcome [32,36,37].

Nevertheless, in HepG2 hepatocellular carcinoma and Madin-Darby canine kidney (MDCK) cells, a direct *KLF4*-mediated downregulation of *CDH1* was observed, downstream of hepatocyte growth factor (HGF, also known as scatter factor/SF)-induced cell scattering (Fig 2). Moreover, *KLF4* expression was activated by early growth response-1 (*EGR1*) under stimulation of HGF and sustained itself through a transcriptional auto-activation loop [38]. HGF-mediated cell scattering is largely dependent on *SNAI1* activation, downstream of HGF signaling [39], implying an important EMT transcription factor in this process. These apparent contradictions might be explained using a contextual view on these cellular events. *KLF4* is able to act as an activator or repressor of downstream genes, depending on the availability of co-activators or co-repressors in its environment.

*KLF5* activates *CDH1* expression and downregulation of *VIM* in non-small cell lung cancer cells [40]. Both *KLF4* and *KLF5* show competition for promoter binding sites with antagonizing effects regarding proliferation [41]. In intestinal cells, *KLF4* represents a marker for differentiated villus cells whereas *KLF5* positively regulates proliferation in the crypt cells [41]. The latter has been confirmed by providing evidence of increased cyclin-D1 (*CCND1*) transcription, colony formation and cell growth in normal ileal cells (IEC-18) and Immorto-Min Colon Epithelial (IMCE) cells overexpressing *KLF5* [42]. Contrastingly however, the same group demonstrated inhibition of proliferation in *KLF5*-overexpressing DLD-1 colon adenocarcinoma cells. Interestingly, in the context of oncogenic Ras, *KLF5* became significantly downregulated by at least two mechanisms, namely reduced mRNA transcription and proteasomal degradation. Reduced expression of *KLF5* in intestinal cancer when compared to normal epithelium could also indirectly promote EMT by relieving the promotional activity upon the *CDH1* promoter.

*KLF6*, originally identified as a tumor suppressor in prostate carcinoma [43], also activates the *CDH1* promoter [44]. The tumor suppressor function of *KLF6* was further demonstrated in hepatic [45] and gastric cancer [46,47] and linked to a role in cellular differentiation. Several mechanisms leading to *KLF6* loss have been described, including promoter hypermethylation [48], somatic mutations and loss of heterozygosity in prostate [43], gastric [47] and ovarian cancer [49]. Interestingly, an alternative mechanism to downregulate *KLF6* was found to involve the generation of three splice variants (SV1-3) with antagonistic effects on the wild type *KLF6* tumor suppressor function [50]. KLF6-SV1, one of these splice variants, is able to drive breast cancer cells into an EMT-like phenotype, with loss of *CDH1* and increased expression of *CDH2* and *FN1* [51]. This was associated with enhanced metastatic potential, however without significant morphological changes. Expression of KLF6-SV1 was found to indicate poor prognosis in several epithelial cancer types, including breast cancer [51], prostate cancer [52] and lung cancer [53]. Primordial findings on KLF6-SV2 point in a similar direction [45]. The origin of this deregulated splicing activity was traced to a germline single nucleotide polymorphism (SNP) in the *KLF6* allele, generating a novel binding site for the SRFS5/SRp40 splicing factor [50]. HGF-dependent phosphorylation of Akt potentiates KLF6-SV1 signaling through subsequent inactivation of the splicing regulators SRSF3 and SRSF1 [54] (Fig 2).

In breast cancer, *KLF17* was identified as a metastasis suppressor, counteracting EMT in the 168FARN murine breast cancer cell line, normal murine (nMuMG) and human (HMLE) breast epithelium [55]. Knockdown of *KLF17* significantly reduced *CDH1*, *TJP1* and *CTNNB1* expression with concomitant increase of *CDH2*, *VIM* and *FN1*. In a cohort of human breast cancers, *KLF17* was found to be decreased in lymph node positive when compared to lymph node negative tumors, hereby indicating a prognostic value. The authors further identified inhibitor-of-differentiation protein *ID1* as a pro-metastatic regulator downstream of *KLF17*, which becomes expressed in breast cancers due to loss of its repressor (Fig 2). Indeed, an inverse correlation between *KLF17* and *ID1* was noted, specifically high *KLF17* – low *ID1* and low *KLF17* – high *ID1* in node negative and node positive breast tumors respectively. *ID* factors (*ID1* – *3*) are helix-loop-helix (HLH) transcription factors lacking a basic domain and unable to bind cognate DNA sequences. They act as dominant-negative regulators of the basic HLH transcription factor E47 [19,56]. E47 is an EMT promoting transcription factor capable of directly repressing *CDH1* [24]. On a transient level, the formation of a complex with ID1 inhibits E47 from binding the *CDH1* proximal promoter sequence, hereby switching on E-cadherin expression. However, introduction of *ID1* in E47-induced mesenchymal MDCK cells was insufficient to restore E-cadherin transcription or an epithelial phenotype. Moreover, *ID1* expression maintains a stable EMT phenotype and preserves cell viability [57]. The versatility of *ID* proteins is illustrated by their crucial role in early-phase metastatic colonization of the lung [58].

Associations between loss of *KLF17* and reduced survival have been reported for lung and hepatic cancer [59,60]. In HepG2 hepatic cancer cells, *KLF17* was found to be under post-transcriptional regulation by miR-9, implying an oncogenic and pro-metastatic role for this miR through repression of *KLF17* [61].

### 2.2 TGFß-induced EMT

**Fig 3:**
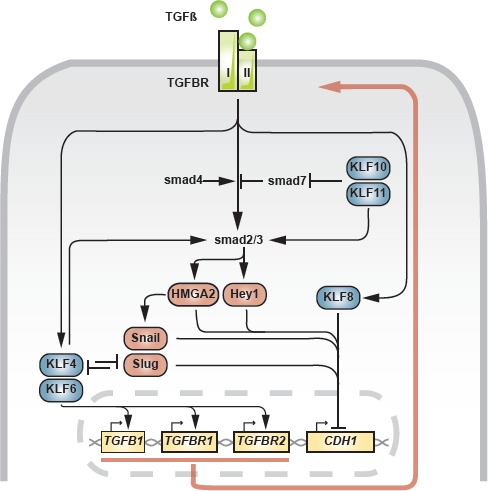
Involvement of *KLFs* in TGFß-induced EMT Blue: *KLF*, red: EMT master transcription factors, yellow: genes. Red arrow indicates positive feedback mechanism.

Epithelial cells can be driven to EMT under the influence of signaling events resulting from upstream extracellular cues (Fig 3). Transforming growth factor ß-1 (TGFß1/*TGFB1*) is known to drive cells towards a mesenchymal state through smad2/3-dependent transcription of *SNAI1* [62,63]. Additionally, HMGA2 and Hey1 have been identified acting downstream of smad2/3 and similarly blocking *CDH1* expression [25,26]. In a mouse model of progressive prostate cancer (*Pten/TP53* null) with stem cell and EMT characteristics, TGFß-induced EMT mainly acted through *SNAI2* and to a lesser extent through *SNAI1* [64] (Fig 3). In this setting, *KLF4* inhibits TGFß-driven EMT by directly repressing *SNAI2*. *KLF4* may sustain a positive feedback loop involving TGFß ligand and receptors through binding of GC-boxes in the proximal promoters, as shown in vascular smooth muscle cells (VSMC) [65]. A similar mechanism had previously been demonstrated for *KLF6* [66].

Given the reciprocal repression between *KLF4* and *SNAI2* [64], the final output generated by TGFß, be it differentiation or EMT, could be determined by the intracellular balance *KLF4*/*SNAI2*. When other channels including signals from the surrounding microenvironment trigger *SNAI2* in excess to *KLF4*, EMT through TGFß-signaling could become the dominant process, sustained by *KLF4*-dependent positive feedback signaling.

*KLF8*, a potent EMT-regulator, is induced by TGFß1 and acts as an indispensable player in the TGFß-mediated EMT in gastric cancer cells [67]. Elimination of *KLF8* led to attenuation of EMT and inhibition of the associated capacity of migration and invasion.

Two members of the *KLF* family, currently known as *KLF10* and *KLF11*, are deeply embedded in TGFß signaling and have originally been named thereafter, respectively TGFß inducible early gene 1 (TIEG1) and 2 (TIEG2) (Table 1). Initially described as directly regulated by TGFß1, *KLF10* [68] and *KLF11* [69] provide supporting actions in the TGFß signaling pathway, exerting anti-proliferative and pro-apoptotic effects in epithelial cells. Both factors show a strong structural similarity, specifically within the zinc finger regions (91%, [69]). In PANC-1, MIA Paca2 and Colo357 pancreatic adenocarcinoma cells, *KLF11* was found to sustain TGFß signaling both by terminating the inhibitory smad7 loop and through activation of smad3 [70,71]. Abrogating effects on smad7 have also been demonstrated for *KLF10* in hepatic and breast cancer cells [72]. Phosphorylation of *KLF11* by MAPK in pancreatic cancer cells abrogates the inhibition of smad7, leading to decreased TGFß-mediated growth inhibition [70]. A transcriptomic screen in TGFß-and EGF-stimulated kidney proximal tubular cells for *cis*-regulatory elements in the differentially expressed gene pool identified, among others, the *KLF*-targeted GC-boxes, and *KLF10* as a principal factor in the EMT-program, mediated by TGFß1 [73]. In agreement, KLF10 protein expression correlated inversely with disease stage in a collection of 95 tissue samples of pancreatic adenocarcinoma and independently predicted progression-free and overall survival in pancreatic cancer [74].

### 2.3 Invasion and metastasis

Local tissue invasion marks the first step of carcinoma progression towards the systemic dissemination of cancer cells and metastatic colonization of distant organs, a multi-step process named the invasion-metastasis cascade [75-77]. Next to important roles of *KLF*s in cell cycle-associated regulation of proliferation (reviewed in [78]), influences on the progression of epithelial cancers towards invasive and metastatic states have been described, often with effects overlapping both fields. This section will describe current knowledge of *KLF*s in invasiveness pathways, apart from direct associations with EMT processes. In addition, upstream triggering events and potential roles of *KLF*s in metastatic dissemination of epithelial cancer cells will be discussed.

**Fig 4:**
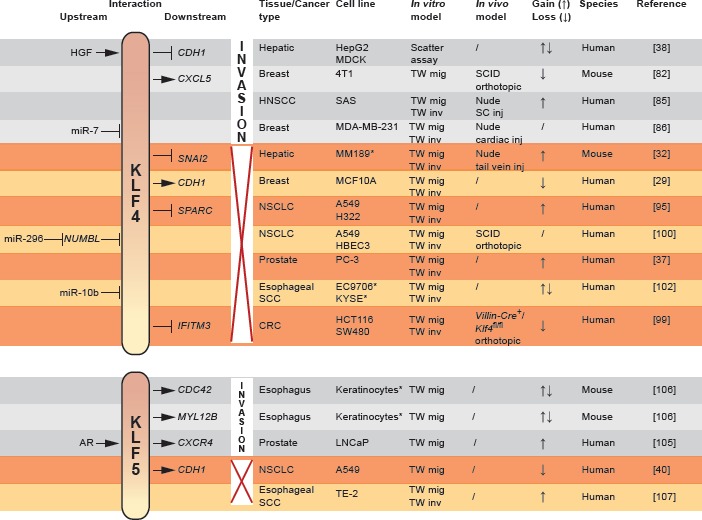
Functional duality in promotion or inhibition (red cross) of invasion by *KLF4* and *KLF5* as dictated by contextual and microenvironmental conditions Upstream and downstream interactions are indicated left and right to the *KLF* symbol respectively, added with experimental modalities and associated references per finding. CRC colorectal carcinoma, HNSCC head and neck squamous cell carcinoma, NSCLC non-small cell lung cancer, SC subcutaneous, SCC squamous cell carcinoma, SCID severe combined immunodeficient, TW transwell. * primary cultures.

Within this scope however, several EMT-modulating *KLF*s regulate invasion. *KLF4* has yielded ambiguous results regarding its oncogenic yet tumor suppressing role (Fig 4). Initially, *KLF4* was identified as a transforming oncogene in oral squamous epithelia [79] and subsequently, increased expression of *KLF4* was found in ductal carcinoma *in situ* of the breast and invasive breast cancers when compared to normal breast epithelium [80]. *KLF4* was a marker of terminally differentiated epithelial cells that became deregulated in dysplastic epithelium. Alternatively, *KLF4* transcription was essential in the maintenance of a breast cancer stem cell (CSC) population [81]. Knockdown of *KLF4* in the MCF-7 and MDA-MB-231 breast cancer cell lines drastically decreased the proportional number of CSC-like cells as defined by *ALDH1* expression, side population and *in vitro* mammosphere formation capacity. Furthermore, downregulation of *KLF4* inhibited breast cancer cell migration and invasion through Notch (*NOTCH1*)-mediated activity. In addition, the same group recently revealed a correlation between *KLF4* expression and production of the C-X-C motif chemokine CXCL5 (*CXCL5*) by primary cancer cells [82]. Chemotactic CXCL5 stimuli recruit C-X-C motif chemokine receptor (*CXCR2*)-positive myeloid-derived suppressor cells (MDSC) to the primary tumor. Interestingly, MDSCs residing at the invasive tumor front express high levels of *TGFB1*, further leading to increased production of CXCL5 in *TGFBR2*-defcient primary breast cancer cells [83]. Although the link between defective TGFß-signaling and CXCL5 expression remains elusive, this mechanism is able to contribute to the chemotactic recruitment of MDSCs. Moreover, enhanced production of CXCL5 by cancer cells gives rise to the systemic secretion of granulocyte/monocyte colony stimulating factor (GM-CSF), a cytokine that contributes to the maintenance of the MDSC pool in the bone marrow. These results may, in part, explain the decreased *in vivo* tumorigenicity and reduced occurrence of pulmonary metastases in a BALB/c mouse model, orthotopically inoculated with stable *Klf4* knockdown 4T1 murine breast cancer cells. The outcome of early-stage breast cancer patients, defined as death by breast cancer, was linked to the immunohistochemical staining pattern of *KLF4* in their primary tumor. Strong nuclear and low cytoplasmic presence of *KLF4* was highly indicative for poor prognosis [84]. Additionally, in squamous cell carcinoma of the head and neck (HNSCC), persistent expression of *KLF4* correlated with poor prognosis, specifically in patients with advanced disease [85]. On a cell line level, ectopic overexpression of *KLF4* increased the tumorigenicity, migration and invasion of HNSCC cells. Corroborating evidence in the CSC domain of breast cancer was established in a study of cancer cell dissemination to the brain [86]. The authors found that, in a mammary CSC population defined as being CD44^+^, CD24^−^ and EpCAM^+^, successful invasion and colonization of brain tissue correlated with high expression of *KLF4* and loss of a microRNA, miR-7, that targets *KLF4* mRNA. This was opposed to CSCs metastasizing to bone, where *KLF4* was expressed at lower levels. In metastatic brain lesions from patients, *KLF4* and miR-7 expression were shown to correlate inversely, indicating a role for *KLF4* transcriptional activity in the establishment of lesions in the brain parenchyma. Interestingly, 9 out of a set of 17 genes previously identified as correlated with brain relapse [87] contain putative binding sites for *KLF4*.

These findings, related to both tumorigenicity and invasive behavior, are in sharp contrast with other reports on *KLF4* function, specifically within the EMT framework as discussed above. Yori and colleagues have extensively described an inverse correlation between expression of *KLF4* and invasiveness, mechanistically based on *CDH1* induction and thus in an EMT context [31]. MCF-10A normal mammary epithelium showed increased migration after knockdown of *KLF4*. In specimens of lung, gastric and prostate cancer, *KLF4* also demonstrated decreased levels of expression when compared with normal tissue counterparts [37,88-92]. Restoration of *KLF4* expression *in vitro* impaired migration and invasion of prostate cancer cells [37]. In the context of epithelial differentiation, the authors have identified the cell cycle inhibitor p27^KIP1^ (*CDKN1B*) as a target of *KLF4*, through which proliferation of pancreatic cancer cells was blocked [93]. *KLF4*-mediated G1/S cell cycle arrest was previously shown to imply activation of p21^CIP1^ (*CDKN1A*) [94]. However, loss of *KLF4* also proved to be stimulating invasion, independent of *CDKN1B*, by the increased deposition of secreted protein acidic and rich in cysteine (*S PARC*, also known as osteonectin) [95]. The involvement of SPARC, a small calcium-binding glycoprotein that modifies cell-matrix adhesion, was already shown in breast cancer and melanoma progression due to its ability to induce *MMP2* and *SNAI1* and repress *CDH1*, hereby promoting ECM invasion and EMT [96,97]. These results coincide with the theory of *KLF4* as a suppressor of EMT and invasion. The antagonistic effect of *KLF4* on tumorigenicity and disease progression was further demonstrated in colorectal carcinoma (CRC). *IFITM3*, an interferon-inducible gene overexpressed in CRC [98], is directly repressed by *KLF4* [99]. Also recently, repression of *KLF4* by the cell polarity protein Numb-like (Numbl/*NUMBL*) was reported from knockdown experiments in the A549 lung cancer cell line. Downregulation of miR-296 causes aberrant expression of its target *NUMBL* leading to reduced *KLF4*-expression and increased random cell migration, invasion and *in vivo* metastasis [100]. This mechanism may be more general since loss of miR-296 is described in several cancers [101]. Another microRNA, miR-10b, had been associated with metastasis by directly targeting *KLF4* [102] or indirectly inducing the expression of *RHOC*, a prominent pro-metastatic gene [103].

In prostate cancer, *KLF5* was found to become expressed downstream of androgen receptor (AR) signaling. The chemokine receptor *CXCR4*, a direct transcriptional target of *KLF5*, is subsequently activated and binding of its ligand CXCL12 (*CXCL12*, also known as SDF-1α) underlies the preferential chemotactic migration of prostate cancer cells to organ sites with elevated levels of CXCL12, for example bone [104,105]. Furthermore, from a study of keratinocyte migration, it is reported that *KLF5* activates the expression of cell-division-cycle 42 (*CDC42*), myosin light chain (*MYL12B*) and their upstream regulator integrin-linked kinase (*ILK*), hereby directly driving cell migration [106]. However, in esophageal cancer cells, *KLF5* inhibited proliferation and invasive behavior [107]. This is in agreement with the more recently identified association between siRNA-mediated loss of *KLF5* and increased expression of *CDH1* in A549 lung cancer cells [40]. Similar to *KLF4*, *KLF5* seems to act in a context-dependent fashion, partially determined by the genetic background (Fig 4).

*KLF8* activates invasion in cooperation with focal adhesion kinase (FAK) by increased transcription of matrix metalloproteinase-14 (*MMP14*, also known as MT1-MMP) in gastric cancer cells (Fig 2). In parallel, nuclear transportation of ß-catenin and expression of T-cell factor 1 (*TCF1*), participate in the initiation of *MMP14* transcription, indirectly effectuated by *KLF8* [108]. In addition, *KLF8* directly activated *MMP9* expression [109]. Conversely, knockdown of *KLF8* drastically inhibited lung metastasis in nude mice. In human breast and gastric cancer, overexpression of *KLF8* has been shown to predict poor prognosis [109,110]. Similar conclusions resulted from a study in hepatocellular carcinoma, attributing a pro-invasive role to *KLF8*, conferring early relapse in human HCC [111].

Emerging data, although still limited, have identified *KLF9* as a marker of differentiation in glioblastoma neurosphere cells. Knockdown of *KLF9* was sufficient to rescue differentiating neurosphere cells as exposed to retinoic acid [112]. Current knowledge points toward a regulatory role of *KLF9* in the proliferation and differentiation of diverse cell types [113,114]. *KLF10* is known as a supportive player in the TGFβ signaling cascade and is able to initiate apoptosis [115]. Next to its role in TGFβ-mediated EMT, *KLF10* repressed the *EGFR* gene through potential binding sites in its proximal promoter. Reduced expression of *KLF10*, as reported for breast and pancreatic cancer [74,116], can promote invasive and metastatic behaviour by enhancing *EGFR* expression [117]. This may be considered in contrast with its supporting activity in TGFß-induced EMT, however depletion of *KLF10* may be substituted by *KLF11*, which exerts identical effects on smad proteins. In a sense, invasion can then be propelled through intact EGFR signaling while preserving the EMT mechanism as driven by TGFß.

## 3 *KLFs* in stem cell transcriptional circuitries

Consequent to their structural properties and involvements in diverse cellular processes like proliferation, differentiation, apoptosis, EMT and motility, the activity of the *KLF* family shows a high degree of context dependence, related to both tissue and cellular backgrounds. A counterintuitive finding would be the absolute requirement of *KLF4*/*Klf4* in somatic cell reprogramming [118,119]. It has been demonstrated that induction of adult somatic cells to pluripotent cells (induced pluripotent stem cells, iPSCs), with characteristics similar to embryonic stem cells (ESCs), can be managed through the introduction of KLF4 (human *KLF4*, mouse *Klf4*), SOX2 (human *SOX2*, mouse *Sox2*), OCT4 (human *OCT4*, mouse *Oct4*) and c-Myc (*MYC*), also referred to as the *Yamanaka* factors. From this set of transcriptional regulators, c-Myc has been regarded the only dispensable factor, leading to a less efficient yet more specific induction process [120]. In these studies, adult fibroblasts, murine or human, are often used as a somatic source to be redirected to a pluripotent state. *Klf4* has been proven necessary for both induction and maintenance of pluripotency and self renewal. Interestingly, *Klf4* is able to bind a distal enhancer of the central reprogramming factor *Nanog* [121], as well as its proximal promoter element [122,123]. Moreover, a mechanism of redundancy between different *Klf*s has been revealed in the maintenance of self renewal capacity. *Klf4*, initially considered as dispensable for sustaining the stem cell state, acts in cooperation with *Klf2* and *Klf5* to regulate the expression of *Nanog* through its distal enhancer element [121]. Only the coordinated and simultaneous depletion of *Klf2*, *Klf4* and *Klf5* drives ESCs into differentiation, indicating an important function of these *Klf*s in phenotype maintenance of embryonic/induced stem cells. In addition, it was noted that forced downregulation of *Klf2*, *Klf4* and *Klf5* gave rise to cells solely expressing typical ectodermal, and not endodermal, markers (*Fgf5*, *Nes*, C*xcl12*), suggesting downregulation of lineage specific genes by certain *Klf*s.

Apart from the identified redundancy, *Klf5* by itself was shown to exhibit specific functions in the commitment of ESCs to an undifferentiated state. Chromatin immunoprecipitation experiments demonstrated binding of *Klf5* on the promoters of both *Nanog* and *Oct4*, and vice versa. *Nanog cis*-regulatory elements were detected in the *Klf5* genomic region, indicating a feedback loop in ESCs [124]. *Klf5* was found to act independently of *Klf4*, underlining its potential importance as a sole factor in maintaining the ESC state. Elimination of *Klf5* impaired maintenance of the undifferentiated state of ESCs and induced the expression of several early differentiation markers. This effect was due to a deregulation of at least eight genes, targeted by *Klf5*, that contributed to the maintenance of ESCs [125].

**Fig 5:**
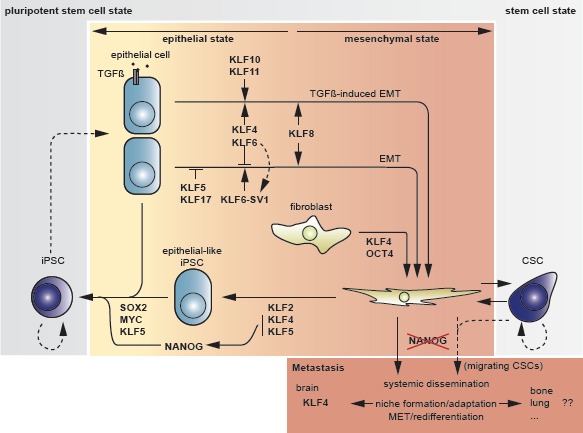
Overview of transitions between cell states and associated actions or counteractions by *KLFs* The center field represents somatic cells, distributed in an epithelial (left) and a mesenchymal section (right) according to their respective properties. The outer field (grey) represents the stem cell state, divided into a pluripotent area (dark grey, left) and a general stem cell area (light grey, right) containing the CSC compartment. The iPSC reprogramming route departs from the somatic fibroblast through the “hypermesenchymal” state via an “epithelial-like” state to iPSC. Dotted arrows indicate putative actions.

Another study uncovered the link between the induction of pluripotency and EMT. Inducing mouse embryonic fibroblasts (MEFs) towards a pluripotent state essentially implied a MET program in the early stage of reprogramming [28,126]. Fibroblasts, showing a mesenchymal phenotype and expressing high levels of *Snai1*, needed the introduction of *Klf4* to activate the epithelial marker E-cadherin (Fig 5). Repression of mesenchymal markers (*Snai1*, *Fn1, Vim*) is mediated by *Sox2* and *Oct4*. The early and temporary transition to an epithelial-like state showed a prerequisite for successful reprogramming of fibroblasts to iPSCs. As mammary epithelial cells highly express endogenous *Klf4* and the initial MET step thus becoming obsolete, the authors showed that reprogramming could be performed by introducing only *Sox2* and *Oct4*. It was therefore postulated that epithelial cells are more amenable to somatic cell reprogramming than their fibroblastic counterparts [127]. Indeed, previous research revealed significantly higher efficiencies using primary keratinocytes instead of fibroblasts [128,129].

Recently, another approach was applied to increase reprogramming efficiencies. By performing sequential, rather than simultaneous introduction of the reprogramming factors, Liu and colleagues obtained a significantly higher yield of iPSCs [130]. The sequential introduction of the *Yamanaka* factors, starting with *Klf4* and *Oct4*, then *Myc* and finally *Sox2* revealed an EMT-MET program in the early phase of reprogramming. *Oct4* and *Sox2* are known to stimulate, respectively inhibit the expression of *Snai2*, a mediator of EMT. Introducing *Sox2* at a later stage during reprogramming allows *Oct4* to regulate an EMT step, hereby possibly homogenizing the fibroblastic cell population to a “hyper-mesenchymal” state [131]. *Klf4* activates *Cdh1* transcription, eventually leading to the subsequent MET step, which is enhanced by the later introduction of *Sox2* with a blocking effect on *Snai2* [130]. Yet, driving *Cdh1* expression is not the only function of *Klf4* as forced expression of *Cdh1* without *Klf4* introduction did not give rise to iPSCs. The early introduction of *Klf4* may be necessitized by its downstream activity upon *Nanog* transcription, as described in murine ESCs, and its ability to prevent ESCs from ensuing a path to differentiation [123]. In a recent study, the heterogeneity among MEFs was considered as another possible cause of the usual poor yield of iPSCs. It was revealed that different MEF subpopulations, based on surface marker expression, showed different degrees of reprogrammability. The CD90^−^/Sca-1^+^ cell populations could be reprogrammed to a pluripotent state using only two of the *Yamanaka* factors, namely *Oct4* combined with either *Sox2* or *Klf4*. From this approach, a partial functional redundancy between *Sox2* and *Klf4* was derived. Moreover, with respect to the intra-population heterogeneity, the rate-limiting MET as observed previously during reprogramming, could be considered only one out of many possible transient stages in a cell between a somatic and embryonic state [132].

## 4 Context dependence and redundancy: lessons from proliferation

Certain studies yielded evidence for pro-tumoral effects, others for tumor suppressing effects by the same *KLF* member (Fig 4). Such duality has been demonstrated for the role of *KLF4* in the regulation of cell proliferation [94]. In an untransformed cell, *KLF4* decreased p53 (*TP53*) levels by repressively binding a PE21 element located in the proximal promoter region of the *TP53* gene. Simultaneously, *KLF4* induced expression of *CDKN1A* leading to cell cycle arrest. The latter effect seemed dominant in untransformed cells, illustrating the tumor suppressor role associated with *KLF4*. Changing the cell's genetic background by introducing an oncogenic Ras^V12^ allele not only abolished the cytostatic function of *KLF4* through bypassing *CDKN1A* induction, but switched it into an oncogenic effect, with *KLF4* showing its dominant action on repressing *TP53* and thus preventing apoptosis. It was concluded that the output mediated by *KLF4* was dependent on the genetic background.

This phenomenon is reminiscent of the dual activity of TGFß signaling on tumor propagation, where TGFß was reported to act as a tumor suppressor in early-stage cancers, and as an oncogene in progressive disease [94,133]. Remarkably, both the TGFß pathway and *KLF4* seem to converge on *CDKN1A* which, on its turn, acts as a gatekeeper preventing oncogenic transformation [94]. *KLF5*, a transcriptional opponent of *KLF4* and a driver of proliferation in intestinal cells [41], was shown to be a key factor in TGFß-mediated inhibition of proliferation. In unstimulated keratinocytes (HaCaT epidermal epithelial cell line), *KLF5* activated cell cycle progression and proliferation by blocking the cell cycle inhibitor *CDKN2B* (p15) [134]. On the other hand, in the presence of TGFß1, *KLF5* became a coactivator in TGFß-induced expression of *CDKN2B*. It was revealed that p300/CBP (CREB-binding protein, *CREBBP*), initially recruited to the Smad2/3/4 complex upon TGFß treatment, reversed the function of *KLF5* by acetylation. In its acetylated state, *KLF5* was able to bind three sites in the proximal region of the *CDKN2B* promoter and activate transcription. Contextual modulation of *KLF5* function was also found in its interaction with *MYC*, a proto-oncogene with proliferation-promoting activity. In similar experiments with keratinocytes, *KLF5* stimulated *MYC* transcription through binding of both a *KLF5*-binding element (KBE) and a TGFß-inhibitory element (TIE) in the proximal promoter region of *MYC*, thus confirming the activating effect on proliferation [135]. Conversely, when TGFß was applied to induce inhibition of proliferation, binding to KBE decreased significantly as *KLF5* was recruited to the TIE sequence by TGFß in order to block *MYC* transcription. These results strongly point to the reversibility of *KLF* transcription function as a tool for balancing between proliferation and differentiation in the maintenance of epithelial homeostasis. This model adds a dimension to the conventional view of changing the levels of different transcription factors in order to mediate opposing outcomes. The dependency of *KLF5*-mediated effects on cellular backgrounds has been further illustrated in intestinal epithelial cells. Ectopic overexpression of *KLF5* indeed stimulated proliferation of normal intestinal epithelium through activation of *CCND1*, yet in colon cancer cells, proliferation and colony formation capacity were reduced through failure of *KLF5* to induce *CCND1* [42]. This tumor suppressing role of *KLF5* has been suggested in clinical breast [136] and prostate carcinomas [137], where loss of *KLF5* was observed when compared to normal tissue counterparts.

Despite being derived mainly from processes related to proliferation, the identified mechanisms comprise molecular modifications that alter the function of the same transcription factor in a context-dependent fashion. These alterations may also be present within an EMT- and/or invasion-related context, mediating cancer progression to a metastatic state. The expression of different *KLF*s is often tissue-specific and existence of redundant subgroups within the *KLF* family is emerging [121,138]. Redundancy enables certain tissue-specific *KLF* family members to exert identical actions in different tissues where expression of companion *KLF*s is limited or absent. Moreover, due to their highly similar *cis*-acting properties combined with *trans*-acting variability, redundant *KLF*s may compete with one another for an identical cognate DNA binding site and impose opposite effects on transcription. *KLF4* and *KLF5* are known for binding site competition as well as for transcriptional autoregulation. *KLF4* activates its own transcription, thus sustaining its presence, and explicitly blocks cell cycle progression [139]. *KLF5*, a stimulator of proliferation, represses the auto-activation loop of *KLF4*. However, *KLF4* is able to inhibit transcription of *KLF5* in order to maintain a dominant role in preventing proliferation [41]. In the intestine, differential expression of both these *KLF*s was demonstrated between crypt and villus cells. Crypt cells with an intense proliferative activity indeed expressed *KLF5* at higher levels, whereas terminally differentiated villus cells showed the opposite, with higher expression of *KLF4*. This implies a *KLF4*/*KLF5* balance shift occurring during the movement of cells from the crypts towards the villi in intestinal tissue homeostasis. Similar implications for *KLF4* in terminal epithelial differentiation have been found earlier in epidermal epithelial cells [140]. This mechanism is engaged by two related *KLF*s in close competition to yield different cellular outcomes, without chemical modulation.

## 5 *KLFs* in metastasis: attempt to an integrated view

The epithelial state is profoundly influenced by the *KLF* family of transcription factors, based on studies using a diverse set of cell types and tissues. As their presence and activity has been demonstrated in different epithelial tissues and derived carcinoma types, added with their fundamental role in somatic reprogramming and its associated transit through an epithelial phase (Fig 5), *KLF*s are thoroughly embedded in a regulatory system that participates in cell fate determination. Nevertheless, the integration of the various mechanisms that have been identified remains a complex challenge.

It has been agreed that the dissemination of cancer cells from the primary site to distant organs and the subsequent successful colonization leading to overt metastasis, requires a significant degree of cellular plasticity [141].

The EMT/MET program and the cancer “stemness” program, provide a dynamic framework in which different *KLF*s appear to contribute a significant role. The presence of both E-boxes as CACCC elements in the promoters of EMT genes allows Krüppel-like transcriptional regulators to participate in the EMT program, in parallel with the previously recognized zinc finger E-box binding and bHLH factors. Moreover, it has been demonstrated that cellular state changes are mediated, in part, by specific *KLF*s in a time-dependent manner. It has become clear that the functioning of *KLF*s, individually and in concert, is dependent of three main determinants: (1) the tissue of origin, (2) the cellular genetic background (context) and (3) the surrounding microenvironment. The final output, as triggered by *KLF* transcription, will often be the result of the interplay between these three features. Furthermore, this also illustrates the intra- and inter-tumor heterogeneity that has increasingly been recognized in several epithelial cancer types [142]. Given the versatility of their functioning, the *KLF* circuitry seems to ft in this plasticity that provides a cancer cell with the ability to proceed to invasiveness and to overcome the multiple barriers towards metastasis.

*KLF8* unambigously drives cancer cells into the mesenchymal state [27], hereby facilitating invasion. However, maintenance of this phenotype is unfavorable in the long term as ectopic survival requires a switch back to a more epithelial state resembling the primary tumor [143]. The contrasting findings regarding *KLF4* and *KLF5* have been shown to originate from both the cellular and microenvironmental contexts. *KLF4* has been reported to have a role in epithelial differentiation and inhibition of growth [29], and thus often becomes repressed in cancerous tissue. Interestingly, the presence of HGF in the proximity of cancer cells, triggers *KLF4* to inhibit *CDH1* transcription [38]. In addition, by modulating splice factor expression, HGF mediated an intracellular shift from the tumor suppressor *KLF6* to the pro-metastatic KLF6-SV1 ([54], see also Fig 2 and 5). These examples illustrate microenvironmental properties igniting invasion and metastasis. An altered genetic background, for example oncogenic Ras, can lead to phosphorylation by extracellular signal-regulated kinase (ERK), hereby inversing the functioning of particular *KLF*s. Within this context, *KLF5* has been shown to lose its proliferation promoting effect that is typical for normal intestinal crypt cells [42]. A similar outcome was observed in normal keratinocytes under exposure to TGFß [134], indicating that Ras-transformed cells have acquired an intrinsic competence to modulate *KLF* function whereas normal cells need the extrinsic influence from the TGFß cytokine. As *KLF5* also directly activates transcription of *CDH1*, downregulation may indirectly play in favor of a progressive phenotype of certain cancer types, but not all. AR signaling was shown to activate CXCR4 protein expression through *KLF5* in prostate cancer cells, propelling chemotactic migration in response to CXCL12, a chemokine abundantly present in the microenvironment and specific sites of metastasis [104,105]. In the latter case, *KLF5* becomes a tool to assist invasive behavior.

TGFß acts as a master regulatory cytokine, being able to manipulate transcriptional functions in early and advanced-stage cancers. Within this scope, TGFß has the potency to induce EMT, partly through *KLF8* [67]. *KLF10* and *KLF11* sustain the TGFß signaling node by inhibiting smad7 and supporting the smad2/3 complex formation. The recent association of low expression levels of *KLF10* with advanced disease in pancreatic cancer [74], may in part be explained by the repressive action of *KLF10* on *EGFR* expression [117]. In a way, TGFß signaling may be maintained by *KLF11*, hereby driving an EMT program, while downregulation of *KLF10* favors the expression of *EGFR*, triggering other pro-invasive signaling cascades. *KLF4* was reported as an opponent of TGFß-induced EMT due to its repression of *SNAI2* [32]. However, as this repression is reciprocal, i.e. *SNAI2* itself represses *KLF4* as well, a balance between both factors can determine a transit towards a mesenchymal state, with a positive feedback on expression of the central TGFß components, mediated by *KLF4*. Similarly, the extensively described tumor suppressor *KLF6* antagonizes EMT, but activates transcription of TGFß ligand and receptors 1 and 2 [66]. In an EMT context, propelled by TGFß, *KLF6* may play a supporting role, favoring cancer cell progression. Moreover, cancer cells with disabled TGFß machinery produce high levels of CXCL5 when confronted with persistent TGFß ligand, both in an autocrine and a paracrine fashion [83]. *KLF4* is also known to activate *CXCL5* transcription [82], corroborating the establishment of a chemotactic gradient that guides MDSC recruitment to the invasive front. As reported most recently, MDSCs induce stemness and tumorigenicity in proximal cancer cells through miR-101-mediated repression of C-terminal binding protein 2 (CtBP2), a corepressor of stemness core genes [144].

When considering the circuitries involving *KLF*s in cancer progression, their involvement in the establishment and maintenance of stem cell phenotypes must not be overlooked. *KLF4*, and *KLF5*, have a fundamental role in the reprogramming of somatic cells and the maintenance of self renewal capability. In this light, it might be speculated that these factors may become upregulated in a subset of cancer cells with stemness properties. This is reasonably supported by the upregulation of *KLF4* in breast cancer stem cells as defined by marker expression, and the observed loss in bulky cancerous tissues. Furthermore, *KLF4* is necessary in the adaptation of metastatic breast cancer cells in the brain niche [86]. On the other hand, EMT confers cancer cells with stem cell properties [145], a finding that apparently contrasts with the indispensable MET step as initiated by *KLF4* during somatic reprogramming [146]. Nevertheless, in order to successfully reprogram cells towards a pluripotent state, *KLF4* needs to be introduced at an early phase, probably in line with its ability to activate *NANOG* and *OCT4* expression, in parallel with *CDH1* [121,122].

**Fig 6:**
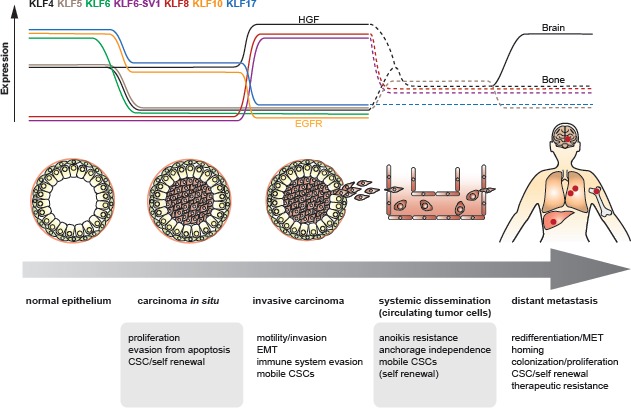
Hypothetic view on the plasticity of *KLF* expression as a contributor to cellular plasticity, shown from normal epithelium through different stages to metastasis Putative expression levels of different *KLF*s vary depending on the requirements per phase. Dotted lines represent putative expression changes based on indicative findings, yet without reported direct evidence. HGF: upregulation of *KLF4* under stimulation of HGF. EGFR: release of EGFR-inhibition through downregulation of *KLF10*. In the metastatic setting, *KLF4* became abundantly expressed in brain metastatic, but not bone metastatic cancer cells.

It must be noted, however, that migrating cancer cells exhibit EMT in combination with self renewal capabilities and anoikis resistance, thus traits of CSCs, yet without pluripotency. Logically, the latter is an unnecessary property as CSCs only need to recapitulate the original epithelial phenotype upon arrival at a distant organ site. Altogether, whether CSCs can be assigned a pluripotent state remains unclear. These features have been merged in a concept involving so-called migrating cancer stem cells [147]. Metastasizing cancer cells should have obtained both EMT and CSC characteristics that enable them to locally invade the surrounding stroma, survive in the circulation and establish metastatic progeny. As *NANOG* represents the cornerstone transcription factor driving cells into a ground pluripotent state [148] and given that *KLF4* and *KLF5* are known *NANOG* inducers, a strict regulation of their expression would be needed to avoid passing irreversibly towards a pluripotent state. However, a certain degree of (transient) expression may be needed to instigate the potential of self renewal. As reported recently, MDSCs are able to contribute to the induction of stemness in cancer cells [144]. Pursuing this hypothetic path, colonization of the metastatic site *de facto* implies proliferation and, therefore, may require downregulation of *KLF5* in Ras-transformed cancer cells. In certain metastatic niches, this may also be the case for *KLF4*, with the preliminary exception for the cerebral tissue environment [86]. As the survival of metastasized cancer cells is dependent on the expression of *ID* genes [58], *KLF17* may need to remain downregulated as well in order to maintain the released repression of *ID1* [55] (Fig 6).

We wish to illustrate the plasticity of *KLF*s in their upstream regulation and their subsequent downstream effects on cellular plasticity as proposed for the progression of cancer to metastatic disease (Fig 4 – 6). This also refects the potential suitability of *KLF*s to be used as markers participating in cancer phenotype definition and adding to the dissection of inter-tumor heterogeneity. As stated recently, future therapeutic approaches against cancers in a metastatic or metastasizing state, will most probably need to comprise a cocktail of compounds interacting with various processes, in an individually tailored strategy [141]. An activity profile containing *KLF*s and downstream target genes may indeed prove to substantially contribute to the individual definition of cancer characteristics at different stages. Furthermore, the gene pool downstream of *KLF* transcription factors can hide novel invasion and metastasis suppressor genes that may be amenable as potential therapeutic targets.
